# Susceptibility of *Angiostrongylus cantonensis* Larvae to Anthelmintic Drugs

**DOI:** 10.3389/fphar.2022.901459

**Published:** 2022-06-21

**Authors:** Daniel B. Roquini, Gabriel L. Silva, Leonardo L. G. Ferreira, Adriano D. Andricopulo, Polrat Wilairatana, Josué De Moraes

**Affiliations:** ^1^ Center for Neglected Diseases Research, Guarulhos University, Guarulhos, Brazil; ^2^ Laboratory of Medicinal and Computational Chemistry, Center for Research and Innovation in Biodiversity and Drug Discovery, Physics Institute of Sao Carlos, University of São Paulo, São Carlos, Brazil; ^3^ Department of Clinical Tropical Medicine, Faculty of Tropical Medicine, Mahidol University, Bangkok, Thailand

**Keywords:** anthelmintic properties, antiparasitic activitiy, Drug Discovery, pharmacology of anthelmintics, helmithiasis, phenotypic screening

## Abstract

Human helminthiasis affects approximately one in five people in the world and disproportionally affects the poorest and most deprived communities. Human angiostrongyliasis, caused by nematode *Angiostrongylus cantonensis*, is a neglected emerging disease with escalating importance worldwide. Chemotherapy is the main control method for helminthiasis, but the therapeutic arsenal is limited. This study aimed to evaluate the antiparasitic and molecular properties of the major available anthelmintic drugs against *A. cantonensis in vitro*. The first-stage larvae (L1), isolated from feces of an *A. cantonensis*-infected rat, were exposed to a set of 12 anthelmintic drugs *in vitro*. The larvae were monitored, and the concentration- and time-dependent viability alterations were determined. From 12 anthelmintic drugs, six (ivermectin, salamectin, moxidectin, pyrantel pamoate, albendazole and levamisole) were identified to affect the viability of *A. cantonensis*. The macrocyclic lactones (ivermectin, salamectin, moxidectin) and the imidazothiazole levamisole, were the most effective drugs, with IC_50_ ranging from 2.2 to 2.9 µM and a rapid onset of action. Albendazole, the most widely used anthelmintic in humans, had a slower onset of action, but an IC_50_ of 11.3 µM was achieved within 24 h. Molecular properties studies suggest that a less lipophilic character and low molecular weight could be favorable for the biological activity of the non-macrocyclic molecules. Collectively, our study revealed that macrocyclic lactones, levamisole, pyrantel pamoate, and albendazole are important anthelmintic agents against *A. cantonensis*. The results of this *in vitro* study also suggest that *A. cantonensis* L1 may be a particularly sensitive and useful model for anthelmintic studies.

## Introduction

Diseases caused by parasitic worms are of enormous human and animal health significance. Human helminthiases are responsible for significant morbidity and mortality in tropical and subtropical countries, especially in poor and marginalized communities with limited access to safe water and adequate sanitation provision. Global estimates suggest that approximately 1.5 billion people are infected with at least one worm (World Health Organization, 2022). Given the critical role these conditions play in global health, the WHO launched in 2021 a new roadmap for neglected diseases for 2021–2030, with strategies to accelerate the control and elimination of helminthiasis and other poverty-associated diseases by 2030 (World Health Organization, 2021a). As highlighted in the WHO roadmap, chemotherapy for at-risk individuals in endemic regions is the main control method for infections caused by helminths. However, there are only a few recommended anthelmintics available for the treatment and control of helminthiasis. Albendazole, ivermectin, levamisole, mebendazole, niclosamide, praziquantel, and pyrantel are available as intestinal anthelmintics in the WHO Model List of Essential Medicines (World Health Organization, 2021b). Other anthelmintics such as fenbendazole, nitazoxanide, pyrantel, and salamectin are widely used as either human or veterinary drugs.

The nematode *Angiostrongylus cantonensis* is known to use multiple rat species as definitive hosts, and this parasite is a common cause of human eosinophilic meningitis (Cowie, 2017). Human angiostrongyliasis has been widely reported from various parts of the world, including several countries in Africa, Southeast Asia, Oceania and South Pacific islands, South America, the Caribbean, the United States, and Europe (Cowie, 2017; Federspiel et al., 2020). In recent years, several cases of angiostrongyliasis in travelers have been reported in the literature, emphasizing the importance of strategies to reduce the emergence of zoonotic pathogens (de Wit and Ricketts, 2021).

Anthelmintics have historically been developed for treatment of non-human parasites (de Moraes and Geary, 2020). Since *A. cantonensis* causes disease in animals and humans and its life cycle can be maintained in laboratory rodents, this species can be an interesting nematode model for anthelmintic studies. Several previous works evaluating the effect of anthelmintic drugs on the motility of *A. cantonensis* have been described in the 1980’s (Terada et al., 1982; Terada et al., 1983; Terada et al., 1984; Terada et al., 1986; Terada and Sano, 1986). However, these studies were done using adult worms. The only previous studies *in vitro* of anthelmintic agents on *A. cantonensis* has been described recently against third-stage larvae (L3) at a very high drug concentration (Jacob et al., 2021) but the activity on first-stage larvae (L1) was not assessed. Using the L1 larvae isolated from feces of an *A. cantonensis*-infected rat, which allows markedly higher throughput than the third-stage larvae and adult worm-based assay, the purpose of this study was to evaluate the antiparasitic activity of the major anthelmintic drugs, including those listed in the WHO Model List of Essential Medicines. The concentration- and time-dependent effects was investigated for all anthelmintic agents. In addition, the molecular properties for the 12 anthelmintic drugs were determined.

## Materials and Methods

### Drug and Reagents

All the anthelmintic drugs, namely ivermectin, salamectin, moxidectin, febantel, pyrantel pamoate, albendazole, mebendazole, fenbendazole, levamisole hydrochloride, nitazoxanide, praziquantel, and niclosamide, were purchased from Sigma-Aldrich (St. Louis, MO, United States) and Cayman Chemical (Ann Arbor, MI, United States). RPMI 1640 culture medium, penicillin G/streptomycin solutions (10,000 units/ml penicillin G sodium salt, 10 mg/ml streptomycin sulfate) were obtained from Vitrocell (Campinas, SP, Brazil). DMSO was obtained from Sigma-Aldrich. On the day of testing, drugs were freshly prepared by weighing and dissolving in dimethyl sulfoxide (DMSO), taking into an account the weight and molecular weight of each compound to reach a stock concentration of 10 mM.

### Larvae Isolation

*A. cantonensis* first-stage larvae (L1), kindly provided by Adolfo Lutz Institute (São Paulo, SP, Brazil), were isolated from Wistar rats (Rattus novergicus) feces according to Rugai's traditional method (da Mota et al., 2020). Briefly, fecal samples collected two months after infection were suspended in RPMI 1640 medium containing 1% (v/v) penicillin/streptomycin solution and centrifuged at 300 x g for 4 min. After the third wash in culture medium with antibiotics, larvae were quantified and transferred to culture plates for anthelmintic assay.

### Antiparasitic Assay

*In vitro* drug testing was performed as previously reported for other anthelmintic assays (Roquini et al., 2019; Porto et al., 2021). Briefly, *A. cantonensis* L1 were transferred to 96-well culture microplates containing 100 larvae/well and were maintained in RPMI 1640 medium containing 1% (v/v) penicillin/streptomycin solution. The initial concentration of the drugs was 50 μM and the compounds that produced an effect superior to 60% after 24 h post-exposure were further serially diluted in medium (50–1.56 μM). The final concentration of DMSO on plates was 0.1% v/v, and wells with the highest concentration of DMSO in medium served as controls. Each drug concentration was tested at least in triplicate, and the experiments were repeated three times. The viability of larvae was scored immediately after adding the drug (time 0) and after 2, 6, 12, and 24 h using an inverted microscope (BEL Engineering INV 100, Monza, MB, Italy) equipped with a BEL Engineering ultra-high definition (UHD) camera and with a 48-inch 4K-UHD monitor system (LG Electronics, Taubaté, SP, Brazil). The larval motility was scored (effect ≥ 60%) on a scale of: 1 (immotile), 2 (intermittent shaking of the head or tail region), 3 (sluggish and motile), 4 (highly active and motile).

### Molecular Properties

The molecular properties for the 12 anthelmintic drugs were calculated using the ADME QSAR module of StarDrop® (Optibrium) and the Compound Activity Workflow of MetaDrug® (Clarivate).

### Statistical analysis

Each assay was performed in triplicate (100 larvae for each replicate, giving a total of 300 larvae for each concentration tested or control) and repeated at least three times on different days. For each phenotypic assay, the mean response and standard error of the mean at each drug concentration were calculated by averaging across worms. For the larval motility score the numbers of larvae were counted for each drug concentration and the percentages of larvae were calculated. Sigmoidal concentration-response curves were calculated in GraphPad Prism software 8.0 and the concentration required to reach 50% inhibitory concentration (IC_50_) values with respective 95% confidence intervals were determined as described previously (Guerra et al., 2019; de Brito et al., 2020).

## Results

### Concentration- and Time-Dependent Effects of Anthelmintic Drugs

**FIGURE 1:**
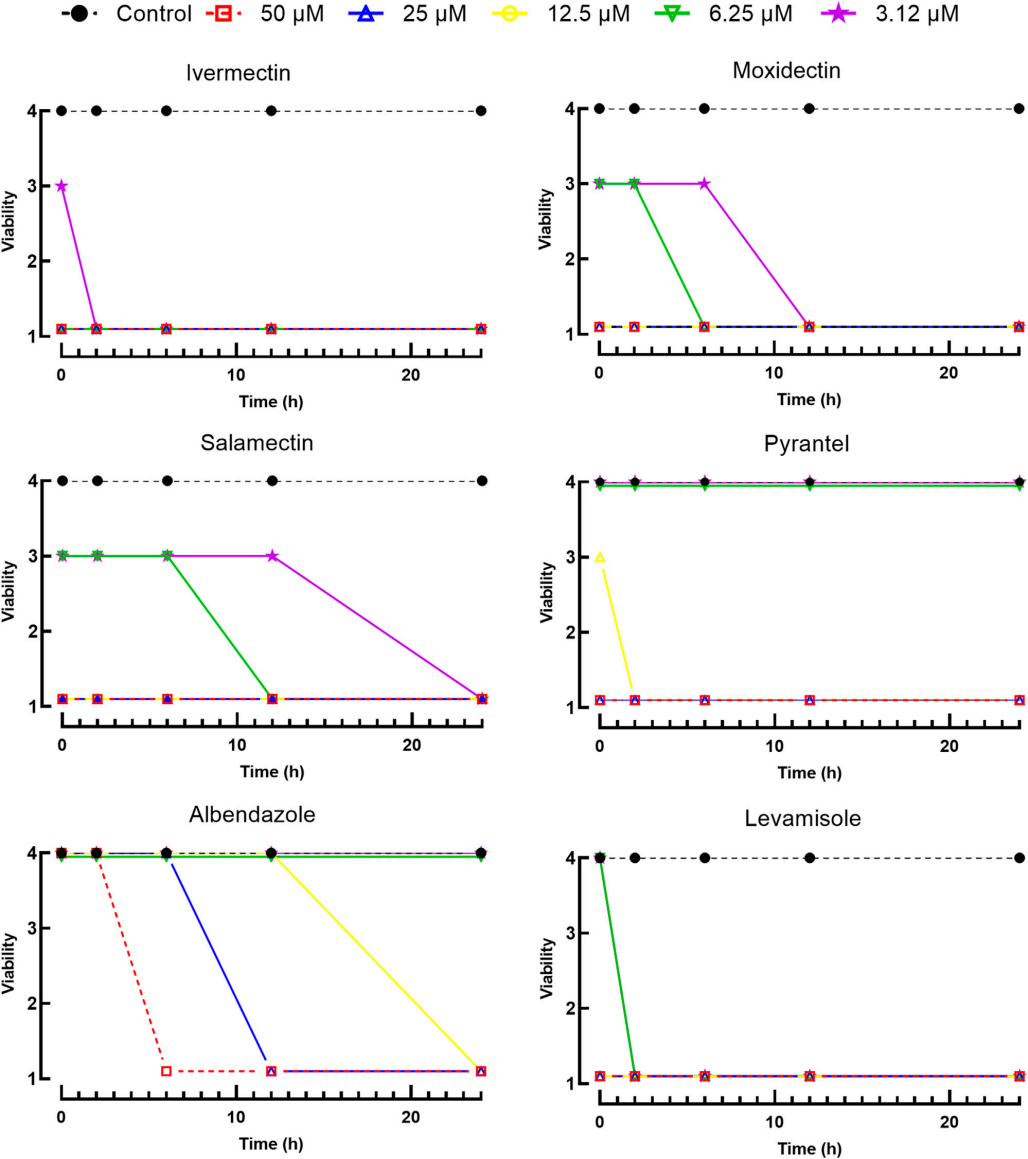
Viability of *A. cantonensis* L1 following incubation with anthelmintic drugs. Larval viability was scored at indicated time points as 1 (immotile), 2 (intermittent shaking of the head or tail region), 3 (sluggish and motile) or 4 (highly active and motile). Data points represent at least three independent experiments conducted in triplicate (each replicate contains 100 larvae).

**FIGURE 2:**
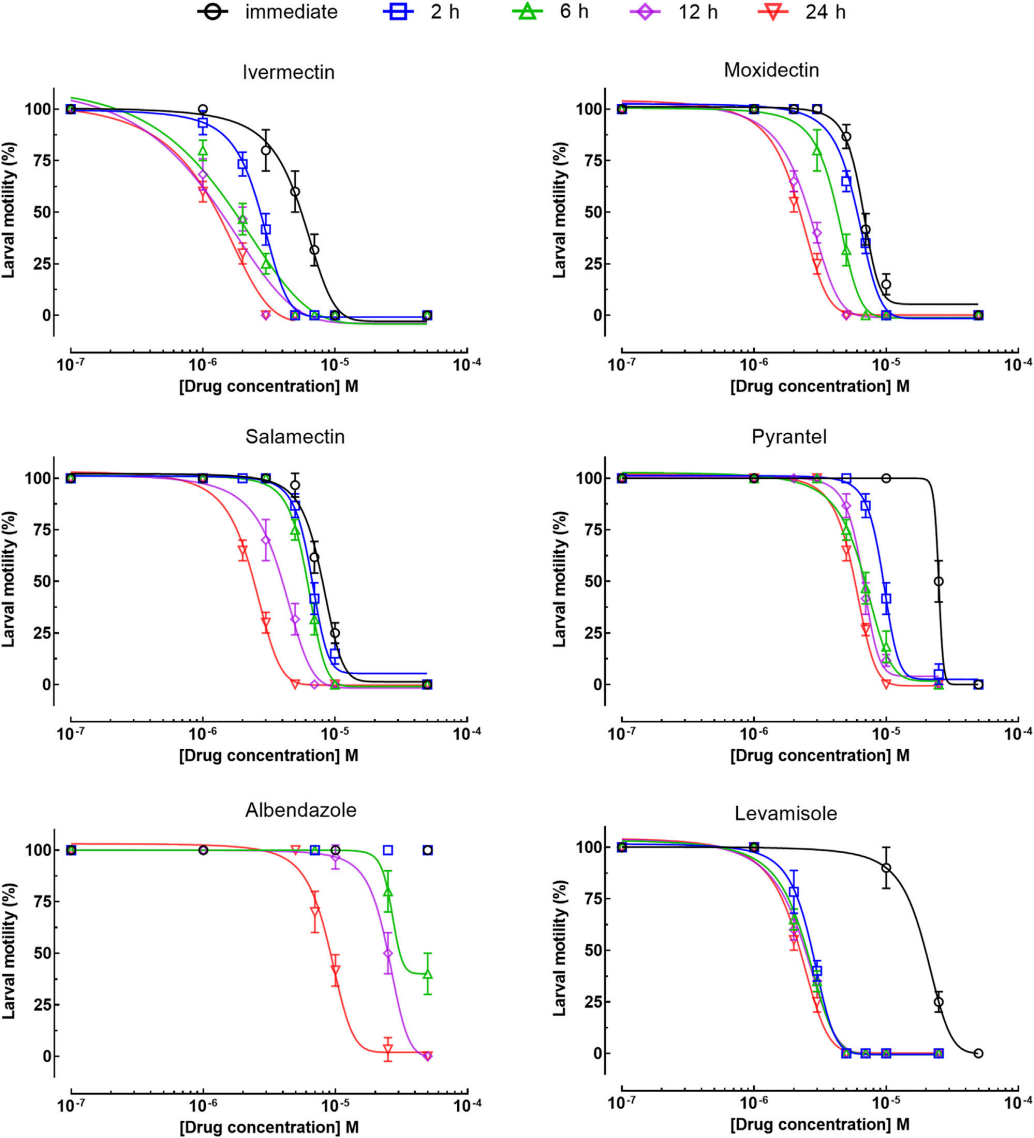
Concentration-response curves for inhibition of *A. cantonensis* L1 motility following incubation with anthelmintic drugs. Inhibition of the motility (score 1) was determined at indicated time points. Data points represent at least three independent experiments conducted in triplicate (each replicate contains 100 larvae), presented as the mean ± standard error of the mean.

The larval motility assay is currently the method of choice to evaluate drug sensitivity of different nematode species (O’Neill et al., 2016; Buchter et al., 2021). Of all the drugs tested at 50 µM, six (ivermectin, salamectin, moxidectin, pyrantel pamoate, albendazole, and levamisole) were identified to affect the viability of the larvae, and these compounds were further tested at a range of concentrations. As shown in Figures 1, 2, all drugs have concentration- and time-dependent effects. Comparatively, macrocyclic lactones (ivermectin, salamectin, moxidectin) and levamisole induced greater immobility than the others. We took ivermectin as an example, which was the anthelmintic drug that had a very fast onset of action on *A. cantonensis* L1, at a concentration of 3.12 μM, the drug caused paralysis of the larvae in the first 2 h and, when the concentration was increased to 6.25 μM, the worms immediately became motionless. At 6.25 μM, the worms were immobilized when exposed to levamisole, moxidectin, or salamectin after 2, 6, and 12 h, respectively. A slightly slower onset of action was observed when parasites were exposed to pyrantel pamoate or albendazole, with a maximum loss of spontaneous movement at 12.5 μM within 2 and 24 h, respectively. For comparison, temporal analyzes of different drug concentrations on larval motility (score of 4 a 1) are shown in Figure 1, whereas concentration-response curves based on the immobile larvae (score 1) are shown in Figure 2. Half Maximum Inhibitory Concentration (IC_50_)A summary of the results of the consolidated IC_50_ values for all six effective drugs on *A. cantonensis* L1 larvae are presented in Table 1. Overall, levamisole had potent *in vitro* activity, with a IC_50_ ranging from 14.7 to 2.2  µM, for immediate action and by 24 h, respectively, whereas albendazole had the highest IC_50_ values. The three macrocyclic lactones ivermectin, moxidectin and salamectin also exhibited potent *in vitro* activity against *A. cantonensis* L1, with a IC_50_ of 1.4, 2.5 and 2.9  µM, respectively, by 24 h. Based on IC_50_ values, the order of loss of spontaneous movement activity for a 24 h treatment period was: ivermectin > levamisole > moxidectin > salamectin > pyrantel pamoate > albendazole. Calculations of several molecular properties showed no significant differences for the physicochemical profiles of the active macrocyclic lactones (Table 2). On the other side, for the non-macrocyclic compounds, the logarithm of the octanol/water partition coefficient for neutral (logP) and ionized compounds at pH 7.4 (logD) were lower for the active drugs, along with lower molecular weight (MW). These findings suggest that a less lipophilic character and low MW could be favorable for the biological activity of the non-macrocyclic molecules. Among the three benzimidazole-containing compounds, albendazole was the only active one (IC_50_ = 11.3 µM). Fenbendazole and mebendazole turned out to be inactive. The calculation of the molecular properties for these three compounds revealed a rather similar pattern. However, one may note that the active compound (albendazole) does not have a second aromatic ring at the left-hand side of the molecule. The lack of this aromatic ring could explain the activity of this molecule in terms of the interaction with the, so far unknown, molecular target(s). Another interesting aspect is that the lack of this aromatic ring lowers the MW of albendazole in comparison with the other benzimidazoles. Low MW is a feature that can be generalized for the other active non-macrocyclic compounds that were investigated herein.

**TABLE 1:** IC_50_ values of ivermectin, salamectin, moxidectin, pyrantel, albendazole, and levamisole on *A. cantonensis* L1.

Anthelmintic	IC_50_ (µM)				
	Immediate	2 h	6 h	12 h	24 h
Ivermectin	5.6 (4.8–6.0)	2.1 (1.8–2.4)	1.8 (1.5–2.1)	1.5 (1.0–1.7)	1.4 (0.9–1.6)
Moxidectin	9.2 (8.6–9.6)	8.7 (8.1–8.9)	5.3 (4.8–5.7)	2.6 (2.1–3.1)	2.5 (2.0–2.8)
Salamectin	10.5 (10.1–10.9)	9.9 (9.6–10.2)	8.5 (8.3–8.9)	5.7 (5.4–6.1)	2.9 (2.3–3.1)
Pyrantel	18.8 (18.3–19.7)	11.4 (10.9–12.1)	10.5 (10.2–11.4)	9.7 (9.2–10.4)	9.0 (8.5–9.5)
Albendazole	>50 (ND)	>50 (ND)	44.1 (42.2–46.7)	21.9 (20.7–23.3)	11.3 (10.1–12.0)
Levamisole	14.7 (13.7–16.1)	2.8 (2.6–3.0)	2.6 (2.4–2.9)	2.4 (2.2–2.6)	2.2 (2.0–2.3)

Data represent the IC_50_ values to loss spontaneous movement (score 1) at indicated time points. 95% CI, ranges are shown in parentheses. Values are calculated from three experiments, and each experiment was performed with three replicates (each replicate contains 100 larvae).ND, not determined.

**TABLE 2:** *In vitro* activity and molecular properties of anthelmintic drugs against *A. cantonensis* L1.

Drug	IC_50_	logS	logS pH 7.4	logD	logP	MW	HBD	HBA	TPSA	Flex	logD heat map
Ivermectin	1.4	0.14	3.63	3.33	3.33	875.10	3	14	170.10	0.12	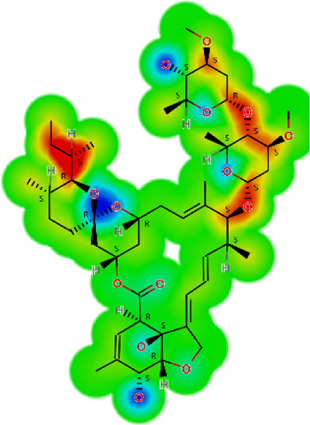
Moxidectin	2.5	0.17	2.82	3.89	3.89	639.80	2	9	116.00	0.06	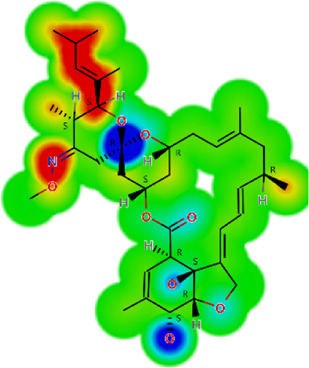
Salamectin	2.9	−0.34	2.81	3.86	3.86	770.00	3	12	154.70	0.07	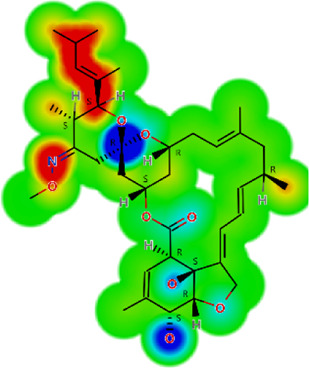
Levamisole hydrochloride	2.2	3.34	1.39	0.27	1.84	204.30	0	2	15.60	0.06	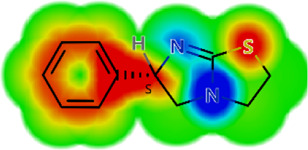
Pyrantel pamoate	9.0	3.83	2.73	−0.33	2.18	206.30	0	2	15.60	0.13	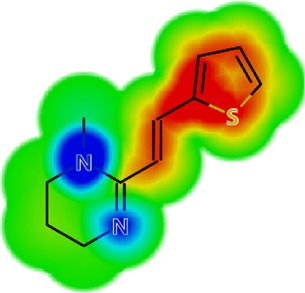
Albendazole	11.3	1.97	3.72	0.39	2.03	265.30	2	5	62.72	0.32	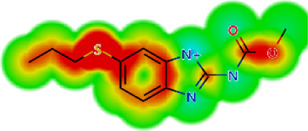
Febantel	Inactive	1.22	4.28	1.70	2.45	446.50	3	10	127.30	0.41	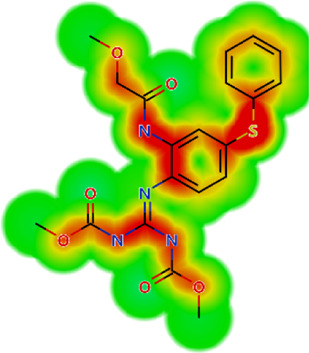
Mebendazole	Inactive	1.52	3.17	0.23	1.70	295.30	2	6	79.79	0.21	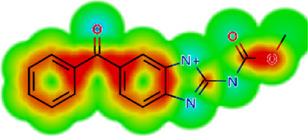
Fenbendazole	Inactive	0.83	3.85	1.01	2.57	299.30	2	5	62.72	0.22	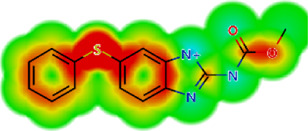
Nitazoxanide	Inactive	2.60	3.20	2.32	2.32	307.30	1	8	114.10	0.27	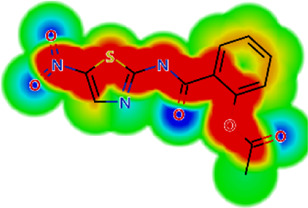
Niclosamide	Inactive	0.69	2.49	3.93	3.93	327.10	2	6	95.15	0.18	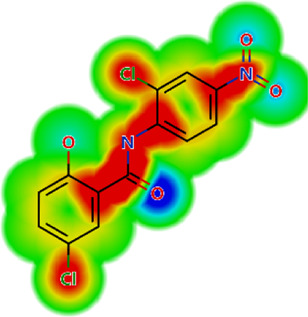
Praziquantel	Inactive	2.86	0.66	2.96	2.96	312.40	0	4	40.62	0.08	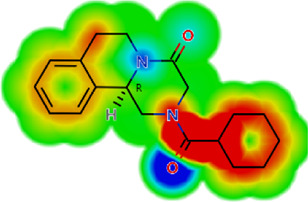

LogS, logarithm of aqueous solubility; logS pH 7.4. logarithm of aqueous solubility at pH 7.4; logP, logarithm of octanol-water partition coefficient; logD, logarithm of organic phase-aqueous phase distribution coefficient at pH 7.4; MW, molecular weight; HBD, hydrogen-bond donors; HBA, hydrogen-bond acceptors; TPSA, topological polar surface area; Flexibility. ratio of rotatable bonds to total bonds. Heat maps for logD. The red-yellow regions contribute positively to the property and the green regions have no influence.

## Discussion

Infection with helminth parasites causes morbidity in billions of people worldwide. Although chemotherapy is the main control method for these infections, the therapeutic arsenal is limited (Ferreira et al., 2022; Mengarda et al., 2022). In this study, *A. cantonensis* L1 were recovered from feces of infected rat and the susceptibility of parasite to anthelmintic drugs available in the WHO Model Lists of Essential Medicines and other commercially available anthelmintics were determined. Additionally, in order to better understand the common features present in anthelmintic agents, several molecular properties were calculated. From 12 anthelmintic drugs, six ivermectin, levamisole, moxidectin, salamectin, pyrantel pamoate, and albendazole (from more effective to less effective) were identified to affect the viability of *A. cantonensis* L1. These drugs are the major course of treatment against parasitic nematode infections. The only previous systematic investigation *in vitro* of anthelmintic drugs on *A. cantonensis* has been described recently using third-stage larvae (Jacob et al., 2021). However, drugs were tested at a very high concentration (1 mM). Since the WHO establishes that an anthelmintic compound cannot exceed 10 μg/ml to inhibit parasite mobility *in vitro* (commonly ranging from 20 to 50 μM) (Pink et al., 2005; Morais et al., 2021), our *in vitro* data indicate that *A. cantonensis* L1 larvae are a particularly sensitive and useful model for anthelmintic studies. Except for moxidectin and salamectin, all the anthelmintics found to be active against *A. cantonensis* L1 larvae are in the WHO Model Lists of Essential Medicines. Most anthelmintics have a broad spectrum of activity (Geary et al., 2010; Choudhary et al., 2022). However, the usefulness of any anthelmintic is limited by the characteristics of the parasite (e.g., susceptibility of the life stage, or susceptibility to the drugs), and these compounds have specific activities for each species (Geary et al., 2010; Keiser and Häberli, 2021). While the benzimidazoles mebendazole and fenbendazole lacked activity against *A. cantonensis* L1, albendazole showed good activity. Albendazole and mebendazole are the most widely used anthelmintics in humans, with a higher efficacy observed for albendazole (Moser et al., 2017), in line with findings from this model. Results of this study with macrocyclic lactones, levamisole and pyrantel pamoate are also consistent with previously reported results (Keiser and Häberli, 2021), which showed the *in vitro* antiparasitic activity against the larval stage of different nematode species, highlighting the importance of *A. cantonensis* L1 in the field of anthelmintic studies. Since *A. cantonensis* laboratory rodent models do exist, this may be the more logical option for a comparative efficacy study and may address the urgent need for new treatments so that goals set by WHO can be achieved. In *A. cantonensis*, anthelmintics mode of action are unknown. However, in nematodes, the mode of actions and targets for anthelmintic drugs have been described (Geary et al., 2010; Wit et al., 2021; Choudhary et al., 2022). Benzimidazoles are predicted to selectively bind near the colchicine binding site of *β*-tubulin. As cytoplasmic microtubules are critical in promoting glucose uptake, the glycogen stores of the parasites are depleted. Regarding the macrocyclic lactones, the major mode of action is binding to glutamate-gated chloride channel and gamma-aminobutyric acid (GABA). This binding causes an increase in the permeability of the cell membrane to chloride ions (hyperpolarization), leading to paralysis and death of the parasite. Pyrantel is a depolarizing neuromuscular-blocking agent causing longstanding nicotinic receptor activation, resulting in spastic paralysis of susceptible nematodes. With respect to the mechanism of action of levamisole, it probably works by targeting the nematode nicotinergic acetylcholine receptor. In tandem, anthelmintic modes of action are multiple and complex and its effects on *A. cantonensis* need to be clarified. Since the biological and toxicological effects of a drug are direct consequences of its physicochemical profile, molecular properties were calculated for all anthelmintic drugs tested. Interestingly, except for macrocyclic lactones, *in silico* results suggests that the anti-*A. cantonensis* activity may be correlated with lipophilicity and molecular weight; these parameters may be important to facilitate the permeation of the compounds through the parasite’s surface (Lago et al., 2019; Xavier et al., 2020) and, consequently, the interaction with their molecular target(s). Regarding the permeation of the macrocyclic lactones, a recent study using the model nematode *Caenorhabditis elegans* has shown that expression of P-glycoproteins in specific barrier tissues, i.e., the epidermis and the intestine can impact the susceptibility to different macrocyclic lactones (Gerhard et al., 2021). The authors demonstrated that active drug ingestion increases susceptibility to ivermectin considerably and to moxidectin only moderately. These findings increase our understanding on drug barriers and uptake routes for susceptibility. Considering the anthelmintic drugs available in the WHO Model Lists of Essential Medicines that were found to be active against *A. cantonensis*, ivermectin and albendazole are more advantageous because they are relatively inexpensive, well-tolerated and widely available. Indeed, the nicotinic agonists levamisole hydrochloride and pyrantel pamoate have some limitations. Although levamisole is a long-established drug and widely used in some developing countries, it causes serious adverse effects its use is now limited in Europe and North America, whereas pyrantel is mainly available in formulations for animals. Interestingly, a recent *in vivo* study in *A. cantonensis-*infected rat showed that pyrantel pamoate exhibited significant antiparasitic properties at a dose of 11 mg/kg, with a worm burden reduction of 53%–73%, demonstrating the potential of pyrantel for the treatment of angiostrongyliasis (Jacob et al., 2022). In conclusion, in this study we demonstrated that ivermectin, salamectin, moxidectin, levamisole, pyrantel pamoate, and albendazole have *in vitro* anthelmintic properties against *A. cantonensis* L1 in a time- and concentration-dependent manner. Calculation of physicochemical properties for all tested compounds revealed that the *in vitro* activity is correlated with the lipophilicity and molecular weight of the drugs. The results of this *in vitro* study also suggest that *A. cantonensis* L1 is a particularly sensitive and useful model for anthelmintic studies.

## Data Availability

The raw data supporting the conclusions of this article will be made available by the authors, without undue reservation.
